# Population dynamics of *Citrus tristeza virus* (CTV) in single aphid-transmitted sub-isolates of the South African GFMS12 isolate

**DOI:** 10.3389/fpls.2022.1024556

**Published:** 2022-10-28

**Authors:** K. K. Biswas, M. L. Keremane, L. J. Marais, C. Ramadugu, R. F. Lee

**Affiliations:** ^1^ Citrus Research and Education Center (CREC), University of Florida, Lake Alfred, FL, United States; ^2^ Plant Protection, Indian Council of Agricultural Research (ICAR)-Indian Agricultural Research Institute, New Delhi, India; ^3^ Agricultural Research Service, United States Department of Agriculture (USDA), Riverside, CA, United States; ^4^ Botany & Plant Sciences, University of California, Riverside, Riverside, CA, United States

**Keywords:** *Citrus tristeza virus*, single aphid transmission, heteroduplex mobility assay (HMA), serology, genetic marker analysis, biological indexing

## Abstract

Grapefruit trees in South Africa have been cross protected against severe stem pitting genotypes of *Citrus tristeza virus* (CTV) since the 1920s using a mild strain initially called ‘Nartia’ but later referred to as grapefruit mild strain 12 (GFMS12). In the current study, the GFMS12 isolate was used as the source for single aphid transmissions (SAT) using *Toxoptera citricida*, commonly called the brown citrus aphid (BrCA). The BrCA-transmitted CTV sub-isolates were analyzed by the heteroduplex mobility assay (HMA), serological assays, genetic marker analysis (GMA), and selected sub-isolates were biologically indexed. Reverse transcription PCR of genomic regions was conducted using universal primers followed by cloning the PCR products, HMA and sequence analysis; nine genotypes of CTV were identified in the complex of GFMS12, including both severe and mild genotypes. A single BrCA transmitted up to six CTV genotypes simultaneously in one sub-isolate. The HMA was found to be a rapid, reliable tool for the identification of genotypes and can be useful in the development of CTV management strategies and budwood certification programs.

## Introduction

*Citrus tristeza virus* (CTV) is a positive-sense single-stranded RNA closterovirus with a genome of about 20 kb, transmitted by brown citrus aphid (BrCA), *Toxoptera citricida* (Kirkaldy), and other aphid species (Bar-Joseph et al., 1989). CTV is the most economically significant citrus virus worldwide. Symptoms caused by CTV can include stem pitting, seedling yellows, decline on sour orange rootstock, vein clearing, and vein corking, depending on the scion/rootstock combination (Rocha-Pena et al., 1995; Lee and Bar-Joseph, 2000; Manjunath et al., 2000). CTV usually exhibits wide diversity in its nucleotide sequence among different isolates, and often a CTV isolate occurs as a mixture of genotypes in nature, with one genotype usually dominating at any given time (Lee and Bar-Joseph, 2000; Brlansky et al., 2003).

Many CTV isolates have been characterized by their coat protein (CP) gene sequence (Djelouah et al., 2009) and by the 5’ terminal sequence of the viral genome (López et al., 1998). Many full-length CTV genome sequences have been deposited in GenBank. Analysis using several full-length sequences has identified seven major CTV genotypes (Melzer et al., 2010; Harper, 2013): T36 (U16304, Florida isolate causing decline on sour orange), T30 (AF260651, Florida mild isolate), T3 (KC525952, Florida severe isolate), VT (U569902, Israel severe isolate), T68-1 (JQ454870, Florida isolate), NZRB-G90 (FJ525432, trifoliate CTV resistance breaking New Zealand isolate), and HA16-5 (GQ454870, recombinant Hawaii isolate). A comparison of the available CTV genome sequences indicated that the nucleotide sequence is highly conserved at the 3’ terminal 8,400 nucleotide region but shows more diversity in the 11 kb sequence at the 5’ proximal region of the genome (Mawassi et al., 1996).

Several methods have been developed to evaluate the diversity of genotypes within a CTV isolate and to predict the genotype composition: biological indexing to determine the host range (Garnsey et al., 1987; Garnsey et al., 2005), ELISA for detecting mild, decline-causing, and orange stem pitting isolates (Gonsalves et al., 1978; Permar et al., 1990; Nikolaeva et al., 1998), reactivity with strain-specific probes designed based on coat protein sequences (Niblett et al., 2000), comparison of the sequence of the 5’ untranslated region of the viral genomes (López et al., 1998), and genetic marker assays (GMA) (Hilf et al., 2005).

Field isolates of CTV usually contain mixtures of CTV genotypes that differ in biological activities (Dawson et al., 2015). Using single aphid transmissions (SAT), it was demonstrated that field isolates are often a mixture of different CTV genotypes exhibiting diverse biological activities (Broadbent et al., 1996; Brlansky et al., 2003). The presence of a severe CTV genotype capable of causing stem pitting on sweet orange in the BrCA SAT sub isolates of Florida mild isolate T66 has been reported (Tsai et al., 2000). Previous transmission studies using *Aphis gossypii* led to the characterization of T66 as a non-stem pitting isolate on sweet orange. The complex of CTV genotypes were separated from a field isolate using single and multiple aphid transmissions followed by analysis using biological assays and GMA (Brlansky et al., 2003). This study showed that mild isolates often mask the severe isolates which react with monoclonal antibody, MCA13. An MCA13 negative isolate may consist of MCA13-positive genotypes as revealed by SAT studies.

The heteroduplex mobility assay (HMA) is based on genetic differences between viral sequences. HMA was first applied to analyze the relationships of *Human immunodeficiency virus* (HIV) strains (Delwart et al., 1993; Delwart et al., 1995). HMA was used for determining the genetic relationships of *Zucchini yellow mosaic virus* (ZYMV) strains using RT-PCR amplified fragments of the variable regions of the coat protein gene (Lin et al., 2000) or the P1 protein-encoding gene (Lin et al., 2001). The use of HMA resulted in identifying five different genotypes of ZYMV, and the results were further confirmed by sequence analysis.

**Figure 1:**
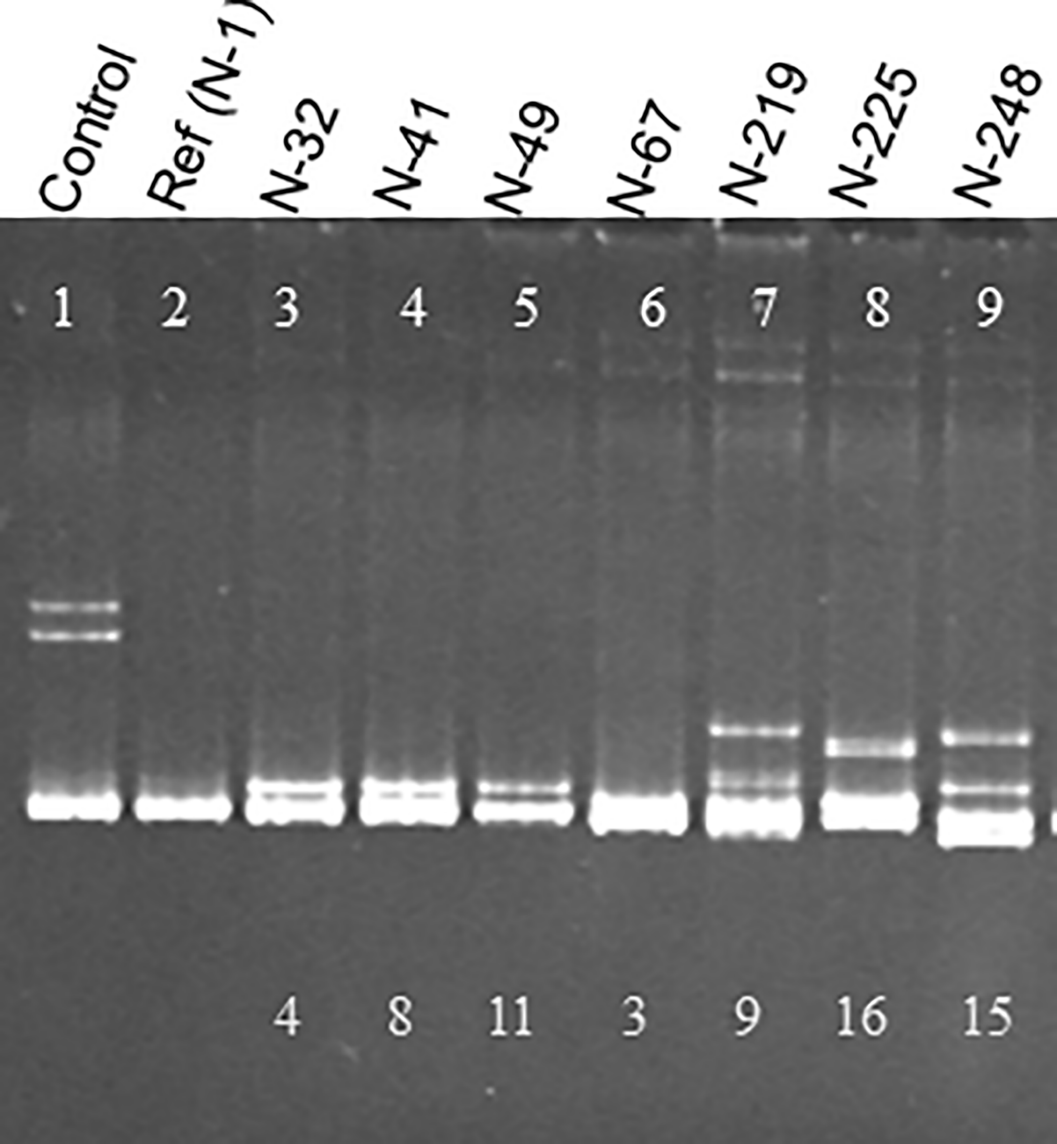
Heteroduplex mobility assay (HMA) showing diversity of CTV populations in South African cross protecting isolate, GFMS12 (B389). A 404 bp region of the CTV genome was amplified by RT-PCR from each sub-isolate using universal primers and cloned. The PCR product from each clone was used for analysis by HMA. The control lane has a mixture of two populations known to differ in sequence by 18%. The PCR product from a reference clone, N-1 was used to analyze a total of 248 clones from B389 by HMA. No heteroduplexes were observed when the clone being analyzed had same sequence as N-1 (lane 2). Seven different HMA patterns were observed as shown here (lanes 3-9). The numbers on the bottom of lanes 3-9 indicate the percent sequence diversity with the reference clone (N-1).

With the HMA, when the RT-PCR products were analyzed on either an agarose gel or a 5-10% non-denaturing polyacrylamide gel, a PCR product with no sequence diversity in the amplified target region moves as a single band. However, when the target region contains a mixture of two or more populations of sequences, multiple bands would be visible on the gels; a single band of homoduplexes, and multiple bands of slower moving heteroduplexes. Heteroduplexes are formed by non-complementary bases resulting in conformational changes which slow down the mobility of the PCR product on the gel (**Figure 1**).

Mild strain cross protection (MSCP) has been an important disease management strategy to protect against severe stem pitting isolates in grapefruit in several parts of the world (Muller and Costa, 1987; Broadbent et al., 1991; van Vuuren et al., 1993). In South Africa, severe CTV isolates cause stem pitting on grapefruit and reduce tree vigor resulting in decreased production and fruit quality. Without the use of MSCP, economic production of grapefruit would not be possible in South Africa (van Vuuren et al., 1993; Marais, 1994). A mild isolate of CTV was selected from a surviving grapefruit tree at the Nartia planting of the 1920s. This isolate, referred to initially as the ‘Nartia’ CTV isolate, was used universally for MSCP of grapefruit and other citrus propagations throughout South Africa for many years. The Nartia CTV isolate was later designated as grapefruit mild strain 12 (GFMS12) (van Vuuren et al., 1993; Marais, 1994; van Vuuren et al., 2000). Over time, the GFMS12 in isolated orchards showed severe CTV symptoms suggesting the original mild isolate of GFMS12 contained more than one CTV genotype (Marais et al., 1996; van Vuuren et al., 2000).

The objective of this study was to characterize the complexity of the mild CTV isolate, GFMS12 using the HMA. The field isolates of GFMS12 were studied previously (van Vuuren et al., 2000; Scott et al., 2013; Zablocki and Pietersen, 2014; Cook et al., 2016; Read and Pietersen, 2016). The GFMS12 isolate used for the current study was maintained since 1994 in the USDA ARS Exotic Citrus Disease Quarantine Facility, Beltsville, MD, and designated as B389. A 404 bp fragment in the 5’ variable region of the CTV genome was selected for analysis of population diversity of CTV genotypes present in the GFMS12 isolate. Further, SAT sub-isolates of GFMS12 were also analyzed by HMA. The HMA technique may be useful in other citrus growing areas for quickly determining the genotypes of CTV present in these areas. This may be valuable for the implementation of citrus certification programs to exclude propagation of severe genotypes in regions where CTV is endemic.

## Materials and methods

### Virus isolates

The Nartia isolate of CTV, designated as the “C” source, was collected in 1971 from a 40-year-old grapefruit tree, cultivar ‘Nartia’, planted near Wellington, Western Cape Province, South Africa (Kotzé and Marais, 1976). The isolate was maintained in a protected greenhouse at the Institute of Tropical and Subtropical Crops, Nelspruit, South Africa, and designated as the source plant for the GFMS12 isolate (van Vuuren and Breytenbach, 2011). In 1994, budwood from this GFMS12 source plant was forwarded to the USDA ARS Exotic Citrus Disease Quarantine Facility, Beltsville, MD, established in a Madame Vinous sweet orange seedling and labeled as CTV isolate B389. The B389 source plant was transferred to the USDA ARS Foreign Disease-Weed Science Research Unit, Ft. Detrick, MD, where it was used as the source plant for single aphid transmissions with the BrCA. The B389 isolate and the resultant eleven aphid transmitted sub-isolates, designated as B389-1 to B389-11, were used in this study.

### Aphid transmission

Aphids (BrCA) from a CTV-free source were transferred to the B389 source plant allowing acquisition feeding for 24 hours. Single aphids were transferred to healthy Madam Vinous sweet orange receptor seedlings enclosed in small mesh bags. After 24 hrs of inoculation feeding, the aphids were removed using a camel hair brush. The plants were sprayed with an insecticide and maintained in a greenhouse with a 16-hour photoperiod and controlled temperatures of 30°C during day and 25°C at night. Samples for serological assays and tissue for RNA extractions were collected six months after aphid challenge. Plants that tested positive for CTV by ELISA were utilized for further analyses.

### Serological assay

Double antibody indirect ELISA was performed using polyclonal anti-CTV antibodies for detection of all CTV strains (Nikolaeva et al., 1995). MCA13 ELISA was performed for detection of decline inducing strains of CTV (Permar et al., 1990). The orange stem pitting CTV ELISA was performed using monoclonal antibodies (Nikolaeva et al., 1998).

### Biological indexing

B389 and two selected SAT sub-isolates, B389-2 and B389-3, were indexed on seedlings of Mexican lime, sour orange, Madam Vinous sweet orange, Duncan grapefruit, and Hamlin sweet orange grafted onto sour orange rootstock, using the standardized host range biological indexing protocol as described (Garnsey et al., 2005). Isolates of B6 (SY-568 from CA), B2 (T30 from FL), and B3 (T36 from FL) were included as reference isolates in biological indexing.

### Genetic marker analysis

Oligonucleotide primer pairs for VT-K17, VT-5’, VT-Pol, T3-K17, T30-Pol, T30-K17, T30-5’, T36-Pol, T36-K17, T36-5’, and T36-CP, were synthesized at Integrated DNA Technologies Inc. (Coralville, IA, USA) and used for GMA (Hilf et al., 2005). The B389 source isolate and the 11 SAT sub-isolates (B389-1 through B389-11) were analyzed using the GMA as described previously (Hilf and Garnsey, 2000; Hilf et al., 2005).

### Universal primers

To detect population diversity of CTV, a region in the 5’ part of the genome where the maximum diversity has been found to exist was identified for this study. An alignment of all sequences available in GenBank were used to design universal primers. A forward primer, CN 488 (5’-TGT TCC GTC CTG SGC GGA AYA ATT), and a reverse primer, CN 491(5’-GTG TAR GTC CCR CGC ATM GGA ACC) were used to amplify a 404 bp nucleotide fragment from the 5’ region (nt 1076 to 1480) of the CTV genome (GenBank accession NC_001661.1). The amplified RT-PCR products were analyzed using the HMA technique.

### RT-PCR

Total RNA from CTV infected bark tissue was extracted using the RNeasy Plant Mini kit (Qiagen, Inc., Germantown, MD USA). Random hexamer primers (0.5µM) were added to 10 µl of the extracted RNA, incubated for 10 min at 85°C, and immediately cooled on ice for 5 min. Reverse transcription (RT) was carried out using MMLV reverse transcriptase (Invitrogen, Inc.; now Thermo Fisher Scientific, Waltham, MA, USA) according to manufacturer’s recommendations. The RT reaction (20 µl) was carried out at 45°C for 1 hr, and the enzyme was heat inactivated at 72°C for 15 min.

One µl of the RT reaction product was used as the template for amplification of multiple targets for conducting GMA (Hilf and Garnsey, 2000). PCR was performed in a thermocycler (Bio-Rad, Hercules, CA, USA) using the following parameters: one cycle of initial denaturation at 94°C for 2 min, 30 cycles at 94°C for 30 sec, (denaturation), 55°C for 45 sec (annealing), and 72°C for 45 sec (extension), followed by one cycle of final extension at 72°C for 10 min. For amplification using the universal primer pairs for HMA, the PCR reaction was performed as described above, with an annealing temperature of 62°C. The PCR products were analyzed on a 1% agarose gel containing ethidium bromide.

### Molecular cloning

The 404-bp amplified products from the RT-PCR conducted using the universal primers (CN488 and CN491) were purified using the QIAquick PCR purification Kit (Qiagen). The purified DNAs were ligated into a *p*Gem-T Easy Vector System I (Promega, Inc., Madison, WI, USA), and *E. coli* (DH5α) cells were transformed following the manufacturer’s recommendations.

### PCR amplification from the transformed *E. coli* colonies

Single colonies were picked from the transformation master plate and placed in 50 µl of colony extraction buffer (Tris-HCl, pH 8.0 containing 2mM EDTA and 1% Triton X-100), and boiled for 5 min. Two to five µl of this DNA extraction was used as a template for a 50 µl PCR reaction to amplify the 404-bp fragment.

### Heteroduplex mobility assay

For HMA, 4.5 µl aliquots of PCR amplified products of two clones (a reference clone and a test clone within each sub-isolate) were combined, and 1 µl of 10X annealing buffer (100 mM Tris-HCl, pH 8.0 containing 1M NaCl and 20 mM EDTA) was added. The DNA mixture was denatured at 95°C for 10 min, reannealed at 68°C for 1 hr, incubated at 0°C for 10 min, and then electrophoresed on a 10% polyacrylamide gel (Bio-Rad ready gels) for 2.5 hrs at 120 volts at 4°C. Heteroduplexes, when present, migrate between the lower band of homoduplex DNA and the upper band of ssDNA. The heteroduplexes indicate that the test clone is genetically different from the reference clone (**Figure 1**). The first clone picked from a transformation plate for each sub-isolate was used as the reference clone for analysis of all other clones.

### Nucleotide sequencing and analysis

Representative clones showing heteroduplex bands in the HMA from B389 and its SAT sub-isolates were sequenced in the forward direction using the vector-based M13 primer following standard protocols (Sambrook et al., 1989) at the DNA Sequencing Core Lab, University of Florida, Gainesville, FL. For sequence determination, 2-3 clones with similar heteroduplex bands were sequenced. Multiple sequence alignments were carried out using the program Clustal W, version 1.6 (Thompson et al., 1997), and nucleotide identity was analyzed using Gene Doc version 2.6.002. The phylogenetic relationships of the B389 source isolate and its SAT sub-isolates, along with select CTV isolates available in GenBank were generated by Neighbor-Joining (NJ) phylogenetic trees using a maximum likelihood parameter of MEGA 6.0 (Tamura et al., 2011). The sequences of seven representative CTV genotypes used in the analyses were T36, T30, T3 T68-1, VT, NZRB-G90, and HA16-5. Three other CTV sequences, SY568 (AF001623, California, recombinant isolate), T2K, and T38K were also included in the analysis. The sequences of T2K and T38 (two populations from a severe grapefruit stem pitting CTV Florida isolate T3800; (Manjunath et al., 2000) were provided by Dr. M.L. Keremane, CREC, Lake Alfred, Florida.

## Results

### Heteroduplex mobility assay

**Figure 2:**
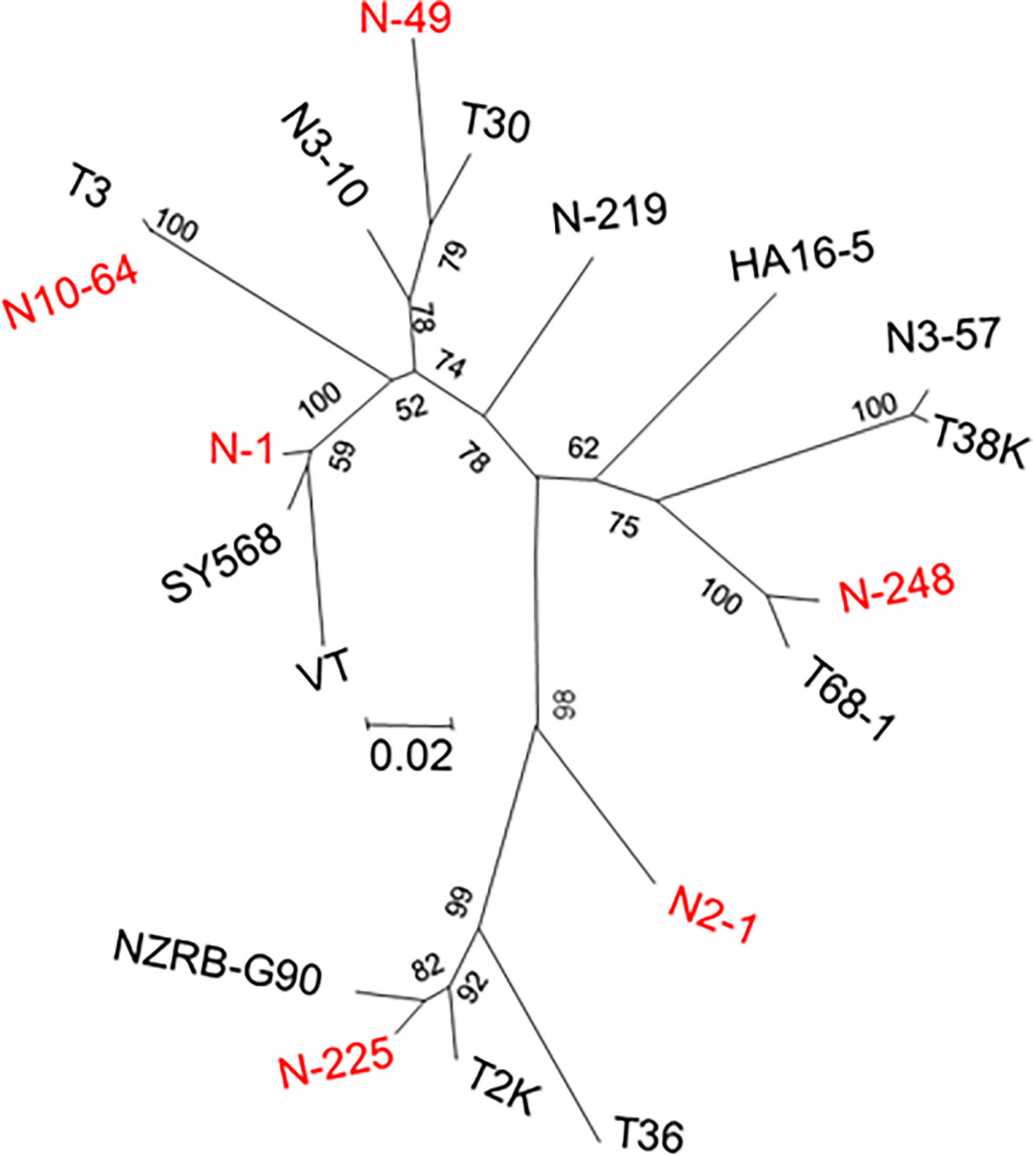
Dendrogram showing the relationships among the CTV populations in the South African isolate, GFMS12 (B389) and single aphid transmitted sub-isolates. An additional ten CTV sequences from GenBank were included in the analysis. All CTV sequence names identified in the source isolate of B389 are indicated by prefix ‘N’. In addition, unique genotypes observed only in sub-populations were, N2-1, N3-10, and N3-57. Sequences were aligned using ClustalX, and Neighbor-joining phylogenetic trees were generated by using maximum likelihood parameter of MEGA 6.0. Bootstrap analysis was done with 1000 iterations, and the values are indicated next to the branches.

A total of 665 clones obtained from the B389 source isolate and SAT sub-isolates were analyzed by HMA. Analysis of 248 clones was conducted using the clone 1 (N-1) as the reference. Eight different HMA patterns were identified (**Figure 1**) and confirmed by sequencing (represented by clones N-1, N-32, N-41, N-49, N-67, N219, N-225, N-248; **Figure 1**). A minimum of three clones showing similar HMA patterns were sequenced. The sequences were compared with a homologous region from seven reference CTV isolates (T36, T30, T3, T68-1, VT, NZRB-G90, and HA16-5) that are available in the GenBank (**Figure 2**). Clones having 5% or less sequence diversity were considered the same genotype. This resulted in identification of five different genotypes from B389 source tree: VT (represented by N-1, N-32, and N-67); T30 (N-41 and N-49); NZRB-G90 (N-225); T68-1 (N-248), and a unique genotype, N-219 (**Figure 1**).

**Figure 3:**
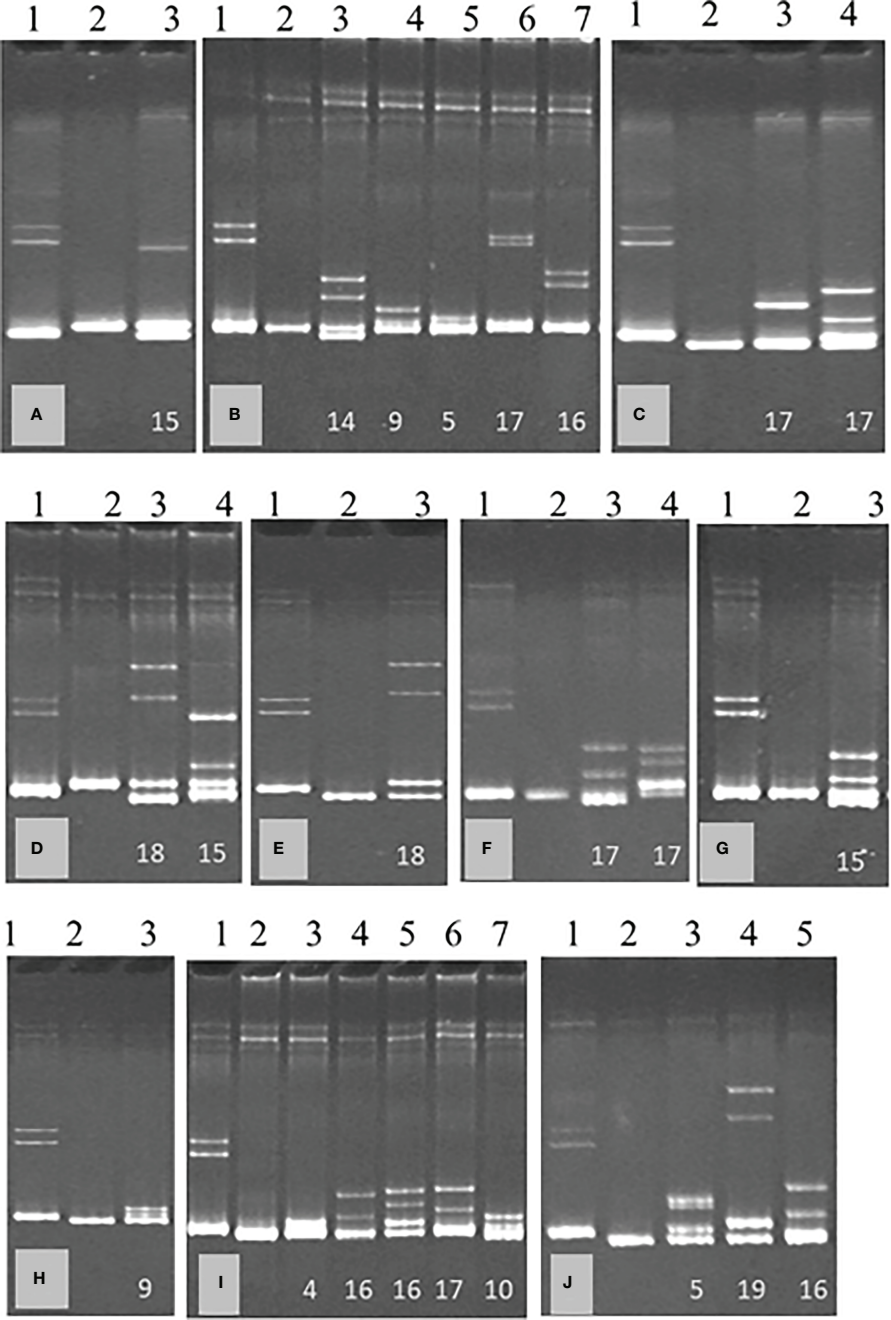
Heteroduplex mobility assay (HMA) of single aphid transmitted sub-isolates of South African cross protecting isolate, GFMS12 (B389); B389-1 **(A)**, B389-3 **(B)**, B389-4 **(C)**, B389-5 **(D)**, B389-6 **(E)**, B389-7 **(F)**, B389-8 **(G)**, B389-9 **(H)**, B389-10 **(I)**, and B389-11 **(J)**. A 404 bp region of the CTV genome was amplified by RT-PCR from each sub-isolate using universal primers and cloned. The PCR product from each clone was used for analysis by HMA. Representative HMA patterns within each isolate are shown. Lane 1 (control) has a mixture of two populations known to differ in sequence by 18%. Clone 1 in each isolate was used as reference isolate for analysis of other clones by HMA. Representative heteroduplexes observed in each sub-isolate are shown in gel images, **(A–J)**. No heteroduplexes were observed in lane 2 in each of the images, **(A–J)** where the clone analyzed was same as reference clone. The numbers on the bottom of lanes 3 onwards indicate the percent sequence diversity with the reference clone.

Clones obtained from SAT sub-isolates B389-1 to 11 were analyzed by HMA. The number of clones analyzed, HMA patterns were observed for each sub-isolate (**Figure 3**), and genotypes identified after genetic analysis of the clones are summarized in **Table 1**.

**Table 1:** A summary of the different CTV genotypes as identified by heteroduplex mobility assay (HMA) followed by sequencing of representative clones with HMA patterns different from the reference clone for each CTV isolate (refer to **Figure 3**).

			Percent of the clones of each CTV genotype								
Isolate	No of clones tested	No of genotypes ;L(HMA patterns) present	N-1(VT-Type)	N-49 T30 type)	N-219	N-225 (NZRB- G90 type)	N-248 (T68-1 type)	N2-1	N3-57	N3-10	N10-64 (T3 type)
B389	248	5 (5)	1	90	1	1	7	0	0	0	0
B389-1	39	2 (2)	0	0	0	9	91	0	0	0	0
B389-2	1	1 (1)	0	0	0	0	0	100	0	0	0
B389-3	38	6 (6)	3	24	0	45	24	0	2	2	0
B389-4	43	3 (3)	84	10	0	6	0	0	0	0	0
B389-5	24	3 (3)	33	0	0	4	63		0	0	0
B389-6	43	2 (2)	36	0	0	0	64	0	0	0	0
B389-7	42	3 (3)	1	26	0	0	73	0	0	0	0
B389-8	40	2 (2)	0	65	0	0	35	0	0	0	0
B389-9	43	2 (2)	0	74	0	26	0	0	0	0	0
B389-10	47	6 (6)	2	38	0	45	2	10	0	0	3
B389-ll	57	4 (4)	5	4	0	0	89	2	0	0	0

The percent of the clones for each genotype present in the source CTV isolate B389, and its 11 single aphid transmitted sub-isolates (B389-1 to B389-11).

The ratios of different CTV genotypes in the B389 source isolate and the eleven SAT sub-isolates are shown in **Table 1**. **Table 2** shows the percent sequence diversity of the 404 bp region in the 5’ end of the CTV genome from nt 1076-1480 (NCBI: U16304) amongst nine different HMA variants observed in this study. The seven reference CTV genotypes mentioned above were also included in this analysis.

**Table 2:** A summary of the percent sequence diversity for all the genotypes found from the CTV source isolate B389 and its 11 single aphid transmitted sub-isolates, from the clones sequenced of the amplified product from RT-PCR using the universal primers as described in Materials and Methods.

CTV Isolate	N-1	N-49	N-219	N-225	N-248	N2-1	N3-10	N3-57	N10-64	VT	T36	T30	T3	T68-1	NZRB-G9	HA16-5
N-1	0	10	8	15	15	15	16	16	9	4	17	8	9	14	16	13
N-49		0	11	18	9	11	7	15	12	12	20	5	12	10	9	13
N-219			0	16	11	13	8	14	12	11	17	11	12	9	15	12
N-225				0	18	8	15	17	16	18	8	16	17	18	2	17
N-248					0	10	14	10	16	17	20	14	17	2	18	11
N2-1						0	15	14	17	18	13	16	17	11	10	15
N3-10							0	16	10	8	18	14	10	14	16	12
N3-57								0	15	8	19	16	15	11	19	12
N10-64									0	11	18	10	0	16	18	13
VT										0	20	9	12	16	18	14
T36											0	18	18	20	8	20
T30												0	10	14	13	13
T3													0	16	13	13
T68-1														0	12	11
NZRB-G90															0	18
HA16-5																10

*The multiple sequences alignments were carried out using the program Clustal W, version 1.6 (Thompson et al., 1997) and nucleotide identity of the 404nt fragment from the 5’ region (nt 1076 to 1480) of L-Pro domain of CTV genome was cloned and analyzed using GeneDoc version 2.6.002. Seven reference isolates of CTV were also included in the analysis*.

### Serology

**Table 3** summarizes the serological properties of the CTV source isolate B389 and the SAT sub-isolates B389-1 to B389-11. The source isolate B389, and all the sub-isolates were positive for CTV when tested with polyclonal ELISA, which detects all CTV infected plants. When tested using the MCA13 ELISA (monoclonal antibody MCA13 reacts with CTV isolates causing decline on sour orange in Florida), all isolates were positive except for SAT sub-isolate B389-3. When tested with OSP ELISA, which is reported to react with sweet orange stem pitting CTV, B389 and sub-isolates B389-1, B389-3, B389-4, B389-6, and B389-8 tested negative, and sub-isolates B389-2, B389-5, B389-7, B389-9, B389-10, and B389-11 tested positive, suggesting they contain CTV genotypes which cause stem pitting on sweet orange.

**Table 3:** Summary of the serological reactivity of B389 isolate and the 11 single aphid transmitted sub-isolates (B389-1 to B389-11) on the polyclonal ELISA, MCA13 ELISA and orange stem pitting (OSP) ELISA.

Sample	Polyclonal ELISA	MCA13 ELISA	OSP ELISA
B389	Positive	Positive	Negative
B389-1	Positive	Positive	Negative
B389-2	Positive	Positive	Positive
B389-3	Positive	Negative	Negative
B389-4	Positive	Positive	Negative
B389-5	Positive	Positive	Positive
B389-6	Positive	Positive	Negative
B389-7	Positive	Positive	Positive
B389-8	Positive	Positive	Negative
B389-9	Positive	Positive	Positive
B389-10	Positive	Positive	Positive
B389-11	Positive	Positive	Positive

### Biological indexing

The results of biological indexing on the standardized host range are summarized in **Table 4**. The indexing profile for the reference isolates of B6 (SY-568), T30 (B2), and T36 (B3) was similar to the profiles obtained in other indexing tests with these reference isolates (Garnsey et al., 2005). The two selected SAT sub-isolates, B389-2 and B389-3 caused slight stem pitting on sweet orange and Duncan grapefruit indicator plants, such a reaction did not occur with the source isolate, B389. The source isolate B389 was mild on Mexican lime and caused a slight decline on sweet orange grafted onto sour orange rootstock. SAT sub-isolate B389-3 did not cause decline in the sweet orange grafted onto sour orange indicator plants, whereas both B389 and B389-2 did.

**Table 4:** Results of biological indexing of B389, two selected single aphid transmitted sub-isolates, B389-2 and B389-3, and standard isolates on five indicator plants.

Isolate	ML	so	swo	Dun Gft	Swt/SO
B389	1	0	0	0	0.5
B389-2	1.5	0	0.5	0.5	1
B389-3	0.5	0.5	0.5	0.75	0
B6 (SY-568)	3	3	3	3	3
B2 (T30)	0.5	0	0	0	0
B3 (T36)	1.5	1	0	0	0.5

Indicator plants used were: Mexican lime (ML), sour orange (SO), sweet orange (SWO), Duncan grapefruit (Dun Gft) seedlings, and sweet orange grafted on sour orange (Swt/SO). Indexing results are indicated on a scale of 0-3 (zero shows no reaction and 3 shows severe reaction).

### Genetic marker assay

The GMA profiles were obtained for the B389 source isolate and its SAT sub-isolates (**Table 5**). Source isolate B389 and SAT sub-isolates B389-3, B389-5 and B389-10 contained a mixture of T30, T36, and VT genotypes. The sub-isolates B389-4, B389-6, B389-8 and B389-11 had a mixture of VT and T36 but not T30, whereas sub-isolates B389-7 and B389-9 contained a mixture of VT and T30 but not T36. Sub-isolate N1, contained only VT genotype. Neither B389 nor any of its SAT sub-isolates contained the T3 genotype.

**Table 5:** The RT-PCR reaction profiles from the genetic marker analysis (GMA) of the B389 source isolate and its single aphid transmitted sub-isolates (B389-1 through B389-11).

Sample	VT-K17	VT-5’	VT3	T3-K17	T30-Pol	T30-K17	T30 5’	T36-K17	T36-5’	T36-Pol
B389	–	–	+	–	+	–	–	+	+	+
B389-1	–	–	+	–	–	–	–	–	–	–
B389-2	ND	ND	ND	ND	ND	ND	ND	ND	ND	ND
B389-3	–	–	+	–	+	–	–	–	+	+
B389-4	–	–	+	–	–	–	–	+	–	+
B389-5	–	–	+		+	–	–	+	–	+
B389-6	–	–	+					+	–	–
B389-7	–	–	+		+	–	–		–	–
B389-8	–	–	+					+	–	+
B389-9	–	–	+	–	+	–	–	–	–	–
B389-10	–	–	+	–	+	–	–	–	–	+
B389-11	–	–	+	–	–	–	–	–	+	-

-: no PCR amplification.

ND: Not done.

Primer sequences used for the reactions were identical to published information (Hilf et al., 2005).

## Discussion

The HMA used in this study was shown to be a rapid and reliable tool for quick identification of the prevalent genotypes. Using HMA, populations having greater than three percent sequence diversity were easily identified. Isolate B389, and the SAT sub-isolates had at least nine different CTV genotypes with six to 19% sequence diversity as revealed by HMA and subsequent sequence analysis (**Tables 2**, **3**). These genotypes included both severe and mild genotypes of CTV, including T30 (mild), VT/SY568 (Israeli/California severe), T2K (stem pitting), T36 (decline on sour orange), T38/T68-1 (stem pitting), T3 (Florida decline/severe), and RB (Resistance breaking) types. In addition, three new genotypes not previously reported: N-219, N2-1, and N3-10-57 were also observed (**Table 3**). In this study, an apparent mild virus isolate was shown to consist of more than nine genotypes. However, since this study was based on analysis of a 404 bp region, some of the variants may be part of defective RNAs known to be present in CTV. Further studies are needed to understand if all variants have biological significance. Some genotypes were identified in a larger number of clones in many sub-isolates, while others were found in minimal numbers in one or a few sub-isolates.

Analysis of 248 clones from the B389 source isolate may not have provided the complete picture since three new genotypes (N2-1, N3-10, and N3-57) were found in some of the SAT sub-isolates. These genotypes were not detectable from the source isolate. Testing of more clones may have revealed additional genotypes. The relative proportions of the different genotypes present in B389 source and the SAT B389 sub-isolates varied as shown in **Table 1**. The genotype represented by clone N-1 (VT type) constituted 84% of the population in B389-4 even though only 1% of the clones tested in the source isolate belonged to this genotype (**Table 1**).

It was observed that a minor genotype in the B389 source could become the major genotype in a SAT sub-isolate (**Table 1**). For example, N-225 constituted only 1% of the genotype in B389 and about 45% of the total genotypes recorded in sub-isolates B389-3 and B389-10. N-248 in B389 made up 7% of the genotypes, while in SAT sub-isolate B389-5 and B389-7, it constituted 63% and 73% of the genotypes, respectively. Furthermore, it was observed that one genotype may be a minor constituent in one sub-isolate but a major component in another sub-isolate. For example, N-248 was found at 2% frequency in B389-10 but at 91% and 89% frequency in sub-isolates B389-1 and B389-11, respectively.

Serological analyses of B389 and the 11 SAT sub-isolates provided evidence that CTV genotypes segregate upon single aphid transmission (**Table 3**). One sub-isolate, B389-3, did not react with MCA13 in ELISA, whereas the source isolate and all the other SAT sub-isolates did. The biological indexing also revealed that sub-isolate B389-3 did not cause decline in sweet orange grafted onto sour orange rootstock (**Table 3**). The source isolate B389 was negative when tested in OSP ELISA, while the sub-isolates were either OSP ELISA negative (5 sub-isolates) or positive (6 sub-isolates) (**Table 3**).

GMA was used to examine a B389 isolate (B7) from the Beltsville Exotic Disease Collection. The authors do not report the GMA profile for the B389 isolate; they did indicate that from the 14 isolates in the collection originating from South Africa, three isolates had a T3 genotype, nine had a VT genotype, one had a T30 + VT genotype, one had T3 + T36 genotype, and two isolates were not assigned to any standard group. Our analysis of genotypes by GMA revealed that the source isolate B389 and the SAT sub-isolates consisted of a mixture of different genotypes; T30 (mild isolate from Florida), T36 (quick decline strain of Florida), and VT (Israel isolate) (**Table 5**). The sub-isolates also had mixtures of two or three genotypes (**Table 5**). Interestingly, VT genotype was present in all the sub-isolates analyzed by GMA. Comparing the relative occurrence of specific genotypes was not feasible with the GMA technique.

A mixture of different CTV genotypes, T30, T36, and VT were reported in the Florida field CTV isolate, FS627 by using single and multiple aphid transmissions, followed by GMA (Brlansky et al., 2003). Single aphid transmission of B389 also resulted in the discovery of additional genotypes, as revealed by GMA.

The cross-protecting isolate GFMS12 has been studied by using various methodologies. BrCA was used for SAT of GFMS12 isolate and the sub-isolates were analyzed by biological indexing on Mexican lime and Marsh grapefruit. Genotypes with severe variants compared to the source isolate were found in addition to mild parental genotypes (van Vuuren et al., 2000). Restriction fragment length polymorphism (RFLP) pattern and single strand conformation polymorphism (SSCP) of the coat protein gene suggested that some sub-isolates contained more than one CTV strain or genotype. One of the sub-isolates from the above study (van Vuuren et al., 2000), GFMS12-8 was inoculated into Madam Vinous sweet orange, sour orange, Mexican lime, and Duncan grapefruit seedlings (Cook et al., 2016). The infected plants were analyzed by eight strain-specific RT-PCRs and sequencing. In another study, three GFMS12 sub-isolates (12-7, 12-8, and 12-9) generated by SAT (van Vuuren et al., 2000) were characterized by Illumina sequencing. The T68 genotype was shown to be the dominant genotype, with reads of some genomic regions suggesting the presence of other minor genotypes (Zablocki and Pietersen, 2014). In another study of the above three sub-isolates, B165-like sequences were found based on the sequence of ORF1a region, but when p23 region was analyzed, the results suggested dominance by a VT-like genotype.

Field trees cross protected with GFMS-12 isolate were sequenced using Illumina MiSeq technology. These trees had been in the field for 23 years and exposed to natural challenge. The resistance breaking (RB) isolate of CTV was found to be the major component, with minor sequence types such as the Kpg3/SP/T3 groups being the second most prevalent (Read and Pietersen, 2016).

The 3’ half of the CTV genome is known to be more conserved than the 5’ half (Mawassi et al., 1996). In the present study, HMA was conducted with a 404 bp amplified fragment (nt 1076 to 1480) from 5’ half of the CTV genome, which is very diverse. This sequence variation facilitated better identification of viral populations.

The HMA used in this study has been proven to be a rapid and reliable tool for quickly identifying the prevalent genotypes for further molecular studies. Using HMA, genotype populations having greater than three percent sequence diversity were easily identified. B389 source and the SAT sub-isolates had at least nine different CTV genotypes: N-1 (MH615837), N-49 (MH614384), N-219 (MH614385), N-225 (MH614386), N-248 (MH614387), N2-1 (MH614388), N3-10 (MH614389), N3-57 (MH614390), and N10-64 (MH161391) with six to 19% sequence diversity as revealed by HMA and subsequent nucleotide sequence analysis (**Tables 1**, **2**). The numbers in parenthesis refer to Genbank accession numbers. These genotypes included both severe and mild genotypes of CTV: T30 (mild), VT/SY568 (Israeli/California severe), T2K (stem pitting), T36 (decline on sour orange), T38/T68-1 (stem pitting), T3 (Florida decline/severe), RB (resistance breaking) types, and three new genotypes not previously reported: N-219, N2-1, and N3-10 (**Tables 1**, **2**). This is the first report in plant virology of an apparently mild virus isolate having more than nine genotypes.

CTV has become endemic in most citrus industries around the world, and hence the focus of any management strategy would be based on reducing the spread of severe isolates. The results from the present study indicate that HMA can be used to quickly determine the genotypes present in areas where severe CTV isolates present in commercial citrus. HMA can also be used to screen budwood sources for excluding the propagation of severe CTV genotypes. Thus, HMA will be useful in developing management strategies for budwood certification programs to reduce the chance of propagating severe CTV in budwood sources.

## Data availability statement

The datasets presented in this study can be found in online repositories. The names of the repository/repositories and accession number(s) can be found below: https://www.ncbi.nlm.nih.gov/genbank/, MH615837 https://www.ncbi.nlm.nih.gov/genbank/, MH614384 https://www.ncbi.nlm.nih.gov/genbank/, MH614385 https://www.ncbi.nlm.nih.gov/genbank/, MH614386 https://www.ncbi.nlm.nih.gov/genbank/, MH614387 https://www.ncbi.nlm.nih.gov/genbank/, MH614388 https://www.ncbi.nlm.nih.gov/genbank/, MH614389 https://www.ncbi.nlm.nih.gov/genbank/, MH614390 https://www.ncbi.nlm.nih.gov/genbank/, MH614391.

## Author contributions

KB: methodology, investigation, data analysis, original draft preparation. MK: conceptualization, investigation, data analysis, editing. LM: methodology, draft preparation. CR: methodology, editing, data analysis. RL: editing, supervision, funding acquisition. All authors contributed to the article and approved the submitted version.

## Acknowledgments

Help from Tina Gouin, USDA ARS, Beltsville, MD for care of plants and Vern Damsteegt, USDA ARS, Frederick, MD help with aphid transmissions is gratefully acknowledged.

## Conflict of interest

The authors declare that the research was conducted in the absence of any commercial or financial relationships that could be construed as a potential conflict of interest.

## Publisher’s note

All claims expressed in this article are solely those of the authors and do not necessarily represent those of their affiliated organizations, or those of the publisher, the editors and the reviewers. Any product that may be evaluated in this article, or claim that may be made by its manufacturer, is not guaranteed or endorsed by the publisher.
